# Developments in the Biomechanics and Equipment of Olympic Cross-Country Skiers

**DOI:** 10.3389/fphys.2018.00976

**Published:** 2018-07-24

**Authors:** Barbara Pellegrini, Thomas Leonhard Stöggl, Hans-Christer Holmberg

**Affiliations:** ^1^CeRiSM Research Centre “Mountain, Sport and Health,” Rovereto, Italy; ^2^Department of Neuroscience, Biomedicine and Movement Sciences, University of Verona, Verona, Italy; ^3^Department of Sport Science and Kinesiology, University of Salzburg, Salzburg, Austria; ^4^The Swedish Winter Sports Research Centre, Mid Sweden University, Östersund, Sweden; ^5^School of Sport Sciences, UiT – The Arctic University of Norway, Tromsø, Norway

**Keywords:** performance, pole, poling force, ski, skiing technique, track preparation

## Abstract

Here, our aim was to describe the major changes in cross-country (XC) skiing in recent decades, as well as potential future developments. XC skiing has been an Olympic event since the very first Winter Games in Chamonix, France, in 1924. Over the past decades, considerable developments in skiing techniques and improvements in equipment and track preparation have increased skiing speed. In contrast to the numerous investigations on the physiological determinants of successful performance, key biomechanical factors have been less explored. Today’s XC skier must master a wide range of speeds, terrains, and race distances and formats (e.g., distance races with individual start, mass-start or pursuit; knock-out and team-sprint; relays), continuously adapting by alternating between various sub-techniques. Moreover, several of the new events in which skiers compete head-to-head favor technical and tactical flexibility and encourage high-speed techniques (including more rapid development of propulsive force and higher peak forces), as well as appropriate training. Moreover, the trends toward more extensive use of double poling and skiing without grip wax in classical races have given rise to regulations in connection with Olympic distances that appear to have preserved utilization of the traditional classical sub-techniques. In conclusion, although both XC equipment and biomechanics have developed significantly in recent decades, there is clearly room for further improvement. In this context as well, for analyzing performance and optimizing training, sensor technology has a potentially important role to play.

## Introduction

In modern times, from the first 1924 Winter Olympics Games in Chamonix to those in Pyeongchang, South Korea, in 2018, cross-country (XC) skiing is the sport that has probably evolved most, including new race formats, improved equipment and preparation of tracks and extensive changes in technique. In addition to being one of the most physiologically demanding endurance sports (Hoffman and Clifford, 1992; Holmberg, 2015), XC skiing also involves highly complex biomechanics (Smith, 1990). Since propulsive force is produced by the musculature of both the upper and lower body and transmitted to the ground via the skis and poles, XC skiing can be viewed as involving a four-limbed gait, which is rather uncommon for predominantly bipedal humans (Pellegrini et al., 2014).

The traditional classical style includes four different sub-techniques, i.e., diagonal skiing (DS), double poling (DP), double poling with a kick (DK), and the herringbone technique (HB) (Nilsson et al., 2004). During the 1980s, skating, more economical and approximately 10–20% faster than the classical style (Conconi et al., 1983; Karvonen et al., 1987; Pinchak et al., 1987; Fredrick and Street, 1988), was introduced and since 1988, has become an official style for competitive XC ski racing. Skating consists of five different sub-techniques (Holmberg, 1996; Nilsson et al., 2004), between which skiers switch in response to changes in speed and slope and which can, accordingly, be considered to represent a gear system (Holmberg, 1996; Nilsson et al., 2004). Clearly, selection of the appropriate technique may exert an important influence on locomotor efficiency and performance (Kvamme et al., 2005; Andersson et al., 2010; Pellegrini et al., 2013; Stöggl et al., 2018). In fact, XC skiing is still evolving, with both small and more pronounced alterations in existing skiing techniques, as well as development of novel sub-techniques.

The aim of the present perspective was to describe and discuss the major changes in XC skiing in recent decades, as well as potential future developments.

## Evolution of Race Formats and Ski Tracks

**FIGURE 1:**
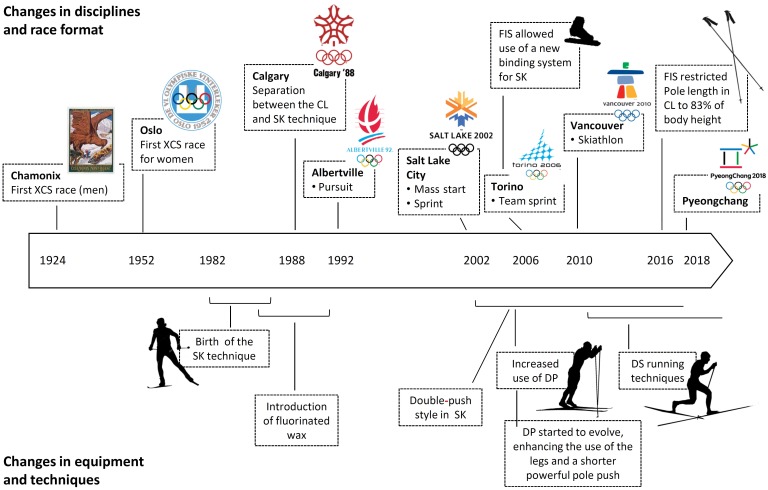
Schematic illustration of the major temporal developments in the equipment and biomechanics of Olympic cross-country skiing. Above, the changes in disciplines; and below, the changes in equipment and techniques. XCS, cross-country skiing; FIS, Fédération International de Ski; SK, skating technique; CL, classic technique; DP, double poling; DS, diagonal stride.

Over the past three decades, several new race formats designed to enhance the popularity of competitive XC skiing have been introduced – the pursuit at the 1992 Olympic Games in Albertville, the mass-start and sprint at Salt Lake City in 2002, the Skiathlon at Vancouver in 2010 and the team sprint at Torino in 2006 (**Figure 1**). A total of 10 of the 12 events involve head-to-head competition, previously associated only with relays. All of these events make great demands on high speed and require extensive alterations in velocity during a race, as well as achievement and maintenance of high finishing velocity (Sandbakk and Holmberg, 2014). Thus, they challenge both technical and tactical competence [e.g., positioning or drafting behind other skiers on flatter portions of the course (Bilodeau et al., 1994)].

According to the manual of the Fédération International de Ski (FIS, 2012), a course should test the skier’s technical and physical abilities while providing smooth transitions between approximately equal lengths of uphill, downhill, and undulating terrain. Recently, tracks are being designed to include multiple shorter laps for better presentation to spectators at the stadium, as well as via television and other media.

The use of snow guns, which began in the 1990s in regions with little snow, expanded in the 2000s and today most World Cup and Olympic XC ski races are held on a combination of natural and artificial snow (E. Macor, personal communication, April 20, 2018) the latter often also being used as a base. The macro- and micro-structures of natural snow are complex and can vary extensively under different environmental conditions (Karlöf et al., 2013). In contrast, artificial snow is generally less variable, providing a harder surface that allows strong pushes without deep penetration by the poles or skis and, furthermore, lasts longer without melting or deteriorating from usage. Snow-grooming machines have also been developed significantly, providing harder and more homogenous surfaces that allow faster skiing.

## Evolution of Equipment

Ekström (1980) has described skiing as “a relationship between man, equipment and environment and all these factors should be adapted to each other to obtain an optimal result.” More formally, a skier’s motion is determined by the balance between propulsive and resistive forces (i.e., aerodynamic drag, gravitational pull while skiing uphill, and friction between ski and snow). Ski-snow friction and drag constitute approximately 30 and 15% of the energy cost, respectively (Spring et al., 1988). Thus, greater power and better economy can be achieved by maximizing propulsive and minimizing resistive forces, a simple fact that has guided the evolution of ski equipment. Because of its complexity, the wide range of speeds (5–70 km/h) and terrain (inclines of -20 to 20%) (Sandbakk and Holmberg, 2014), technique and equipment exert a pronounced impact on skiing.

### Skis and Bindings

From originally being made of wood, since the 1970s XC skis are constructed of polyethylene plastic, fiberglass, and carbon fiber. Olympic skiers have 30–50 pairs (< 25% of which are used in most races) (H-C Holmberg, personal communication, 30 March, 2018), each designed for specific snow temperatures and conditions (Breitschädel, 2012). Sintered thermoplastics have become the standard base material, allowing new processes and treatments that have lowered the friction coefficient substantially (Breitschädel, 2015). At present, 10–15 bases with characteristics specific for various snow conditions are used by elite skiers (Holmberg, 2018). Appropriate preparation of the ski base surface by stone grinding (Breitschädel, 2015) improves gliding substantially. In addition, various glide and grip waxes tailored for different snow conditions further enhance performance. In this context, hydrophobic fluorinated waxes repel moisture, thereby reducing wet friction significantly. During the final ski preparation, various hand-held tools are frequently employed to create different microstructures. In recent years, considerable work and technological development have been devoted to precise characterization of friction during skiing (Breitschädel et al., 2010; Swarén et al., 2014; Budde and Himes, 2017), with the aim of optimizing preparation and waxing. National teams now spend considerable money on highly specialized staff who prepares the skis and all major nations have designated waxing trailers where preparation can be optimized.

For modern skis, the coefficient of friction, which exerts considerable impact on the total mechanical work required (Pellegrini et al., 2014) or energy expended by a skier (Saibene et al., 1989), can be as low as 0.005 on transformed wet snow and as high as 0.035 on cold, fresh snow (Budde and Himes, 2017). During the 50-km freestyle event at the 1992 Winter Olympics, Street and Gregory (1994) observed a significant correlation (*r* = -0.73) between finish time and glide speed. Furthermore, the change in friction due to the texture of a wax or ski base is 0.001–0.010 (Budde and Himes, 2017) and mathematical modeling estimated that lowering the friction coefficient by 0.001 would reduce race time for each kilometer by approximately 2 s (Moxnes et al., 2014).

To improve transmission of the propulsive force of the legs, bindings have been developed to allow more effective control of the skis. Metal bindings were introduced during the first half of the 1900s and a thinner clasp developed in the 1970s. The upper surface of the binding and the boot sole have been shaped to prevent the heel from moving laterally, a necessary constraint for leg pushes when skating.

During the 2005/2006 skiing season, the FIS allowed competitive use of a new binding system based on the clap skate introduced advantageously into ice skating approximately a decade earlier (de Koning et al., 2000; Houdijk et al., 2000). A completely stiff carbon or plastic boot replaces the traditional flexible boot sole and the hinge is beneath, rather than at the tip of the foot, moving the pivot point closer to the ankle joint and shortening the lever arm for more effective leg push-off. In comparison with a conventional system, the skier produces more power that is also more equally distributed over the total push-off, allowing attainment of higher speed over a short distance (Stöggl and Lindinger, 2006). However, this system appears to have no significant effect on skiing economy (Bolger et al., 2016) and probably needs to be adapted further for XC skiing (e.g., with respect to carbon stiffness and pivot point position).

### Poles

Since carbon-fiber alloys and Kevlar wrappings have replaced aluminum as the material for poles, slight changes in design have also occurred. Various ergonomic grips and curved shafts have not proven successful, with apparently little potential for improvement in this connection. A pole shaft with a triangular cross-section introduced recently has a lower moment of inertia during the swing, due to its higher center-of-mass (which allows it to function more effectively as a pendulum), and is also stiffer than the traditional circular shape (Stöggl and Karlöf, 2013) and, therefore, currently most widely used. For application to harder snow, the pole basket has become significantly smaller and is now asymmetric with a diameter of 4–5 cm. Slightly larger ski baskets are sometimes used on new and/or soft snow. To date, no research on the effects of pole basket geometry or size has been reported.

A major challenge with respect to ski poles is achieving sufficient stiffness to apply force efficiently to the track surface. Typical modern racing poles can transmit forces as high as 500–800 N (Swarén et al., 2013b), a value much higher than that normally applied during poling, but which faster skiers can produce at maximal speed (Stöggl and Holmberg, 2011). A major change here involved lengthening classical skiing poles (Nilsson et al., 2003; Hansen and Losnegard, 2010; Stöggl and Karlöf, 2013; Losnegard et al., 2017), which improved oxygen cost (Losnegard et al., 2017; Onasch et al., 2017) and poling mechanics and enhanced peak velocities on both flat and uphill terrain (Stöggl et al., 2010a). However, pole length is limited by recent FIS regulations (see further below).

## Development of the Biomechanics of the Various Skiing Techniques

Overall, the new race formats, which require more rapid acceleration, have altered the earlier goal of cruising at a high, but economical speed throughout the race (Sandbakk and Holmberg, 2014). Consequently, both classical skiing and skating (Nilsson et al., 2004) have been adapted to produce high peak poling and leg push-off forces (Stöggl and Holmberg, 2011), resulting in some development and/or modification, such as the new “kangaroo” or “modern” DP (Holmberg et al., 2005) and double-push skating or “jump” G3 and G4 skating (Stöggl et al., 2008) techniques.

With DP, higher speed requires both higher peak pole forces and poling force impulse (Holmberg et al., 2005). The time available for propulsion at maximal speed is no more than about 0.21 s (Stöggl et al., 2010a, 2011), approximately 50% of the time available at slower speeds (Lindinger et al., 2009) and comparable to the period of contact between the foot and ground while running (Weyand et al., 2010). The legs play an active role in DP as well (Holmberg et al., 2006); during the recovery phase, the ankles, knees, and hip are extended to raise and push the body’s center of mass upward and forward. This process may be so dynamic that the heels and, indeed, sometimes the entire foot are lifted off the ground (Stöggl et al., 2011), making the skier resemble a “kangaroo.” The subsequent downward acceleration of the body by gravity transfers force more effectively onto the poles, complementing the propulsion from upper-body work. This converts the potential energy gained during the recovery phase to kinetic energy (Pellegrini et al., 2014). A more pronounced inclination of the body during the initial phase of poling promotes better skiing economy and may allow a more effective subsequent thrust (Zoppirolli et al., 2015). These revolutionary changes led skiers to employ the DP technique more extensively on a variety of inclines over entire race courses (Stöggl and Holmberg, 2016; Welde et al., 2017; Stöggl et al., 2018).

**FIGURE 2:**
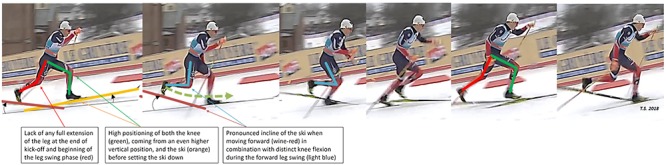
Characteristics of the innovative “diagonal running” XC skiing technique by Johannes Høsflot Klæbo (who won three gold medals in Pyeongchang) during the WC sprint in Drammen.

The resulting challenge to the traditional classical style posed by increased DP utilization, the greater requirement for speed during sprint skiing uphill and the desirability of uphill techniques requiring less grip-wax (and, consequently, allowing better glide on other portions of the course) have all contributed to enhanced utilization of the modified, so-called “running DS technique” on steep uphill terrain. Involving little or no gliding, a higher ski position, more vertical forces at ski plant, and flexed knees during the leg swing, this style allows more rapid cycles, thereby producing rapid acceleration and possibly enhancing ski grip on steep and/or challenging terrain (Stöggl and Müller, 2009). During the final of the men’s classical sprint in the Pyeongchang Olympic Games, the skiers employed the “kangaroo” DP almost exclusively, except for running DS on uphill sections, sometimes called the “Klæbo” style (**Figure 2**).

Skating skiing developed significantly during the first years after it was introduced. The new sprint event demanded faster acceleration and higher speeds, leading to the so-called “jumped G3 double push” skating, resembling a technique employed by inline speed skaters and involving two pushes with the propulsive leg, rather than one on the inside ski edge (Stöggl et al., 2008). During a 100-m sprint, the double-push technique can produce speeds approximately 3–6.9% faster than those reached with conventional skate skiing, with a lower cycle rate as well (Stöggl et al., 2008). Furthermore, on steep inclines the double-push is as fast as the G2 technique, with a lower cycle rate, and faster than the conventional G3 technique (Stöggl et al., 2010b).

Although the extremely high VO_2max_ of world-class XC skiers has not changed since the 1960s, the new sub-techniques require rapid production of force, emphasizing explosive strength and highly developed motor skills (Stöggl et al., 2008, 2011; Lindinger et al., 2009) and today’s elite XC skiers train accordingly (Sandbakk, 2017; Sandbakk and Holmberg, 2017). Better skiers are stronger (Stöggl and Müller, 2009; Stöggl et al., 2010a, 2011), accelerate more rapidly (Wiltmann et al., 2016), possess more lean mass (Stöggl et al., 2010a), and can generate higher peak forces later during the poling phase (Holmberg et al., 2005; Stöggl et al., 2011). Strength has been correlated with starting performance (Wiltmann et al., 2016) and high skiing speed requires extensive involvement of both upper-body and core muscles (Stöggl et al., 2010a; Zoppirolli et al., 2017). Specialists in sprint races are taller and heavier than distance skiers (Losnegard and Hallen, 2014) and competitors in the 50-km classical race in recents Olympic games were heavier (Wood, 2018) than those in the 30-km race in Calgary in 1988 (Norman et al., 1989).

Recently, more focus has also being placed on the downhill sections of a race, where less than 10% of the total racing time is spent, and especially on challenging downhill turns, where faster skiers utilize the accelerating step-turn technique to a greater extent (Sandbakk et al., 2014a,c).

XC skiing competitions for women were introduced at the Oslo Winter Olympic games in 1952, 26 years after the first competitions for men. On the average, women ski 15% more slowly (Sandbakk et al., 2014b) and, in general, compete over shorter racing distances (FIS, 2018), a sex difference unusual for endurance sports. The evolution of female technique has been similar to that of male skiers, although the women’s style appears, in general, to be less dynamic. Sex differences in power production by the upper-body are more pronounced than for the legs (Sandbakk et al., 2014b) and, consequently, the corresponding differences in XC skiing performance have become more pronounced as the contribution by the upper body has risen (Hegge et al., 2016). This consideration also influences selection of technique within a race, e.g., on the same intermediate incline, 50% of the men, but less than 10% of the women utilized DP (Stöggl et al., 2018) and, when skating on uphill terrain, women utilize less G3 (more upper-body involvement) than G2.

## Training

Today’s XC elite skiers perform more sport-specific training than previously, systematically incorporating roller skiing, as well as training of strength, power, and speed into their routines (Sandbakk, 2017; Sandbakk and Holmberg, 2017). Moreover, ski ergometers for upper-body training (Carlsson et al., 2017) and computerized simulation of specific course profiles on treadmills (Swarén et al., 2013a) are utilized by the best elite skiers.

## Role of FIS Regulations

Developments in XC skiing have been influenced, both directly and indirectly, by FIS regulations. In 1984, to prevent this form of skiing and its sub-techniques from disappearing, new rules required that some races be held with the classical technique. More recently, the choice of DP over other classical sub-techniques, not only by world-class, but also less successful XC skiers, poses a new threat.

Consequently, in 2016/2017, the FIS introduced rules limiting exclusive usage of DP. Pole length can be no longer than 83% of body height; on certain uphill sections (i.e., “technique zones”) DP is not allowed; classical racing courses that do not favor exclusive usage of DP are selected (e.g., Oslo, Falun, Val di Fiemme); and track set-up and preparation have been changed (e.g., with a single classical track that follows the “ideal” trajectory and V-boards in curves to prevent extensive usage of lateral skating kicks and/or track changes). Furthermore, stricter surveillance on uphill terrain enforces disqualification for irregular skating strokes.

## Future Perspectives

Despite these extensive efforts to develop ski equipment, there is still considerable room for improvement. New ski bases (e.g., including flour and shorter carbon chains that will not be banned by future EU regulations designed to reduce fluorinated gases) may enhance performance and prolong effective glide. Moreover, there should be improvement and better standardization of stone grinding, as well as of the microstructure applied manually (e.g., pressure and depth), providing better adaptation to various conditions; improvement of skin-skis for challenging snow conditions; further development of the ski-binding-boot unit (e.g., stiffer units to reduce leakage of mechanical energy and, for the Skiathlon, to make the transition between classic and skating easier, more rapid, and safe) and modifications of the poles to increase their resistance to breaking, without adding weight (especially important for head-to-head races).

Evaluation of the gliding properties of skis, still based on field testing using standard methodology (primarily photocells, test pilots, and athletes), always involves the skier’s subjective judgment, but could be made more objective and standardized utilizing sensor technology (Kirby et al., 2018), better management of large datasets, more rapid transfer of test data to practical recommendations (including more powerful statistical analysis), and more effective use of meteorological forecasts. Moreover, technology might also be used to monitor glide/grip and provide better overall evaluation of performance during and following the race.

The significant biomechanical development of skiing techniques over past decades, resulting in higher peak force, power and speed, will continue. Although FIS regulations have apparently reduced exclusive use of DP, thereby preserving the classical technique, future investigations must evaluate which sub-techniques skiers choose in connection with different types of terrain, snow conditions, race formats, and tactical approaches and determine whether these are advantageous in terms of physiological demands and energy expenditure. Such investigations are facilitated substantially by miniaturized GNSS and inertial sensors that can monitor a skier’s speed, motion, and technique (Stöggl et al., 2014) continuously and non-invasively during both training and competition.

## Ethics Statement

This manuscript is based on a review of existing scientific literature and no data involving humans or animals have been acquired directly.

## Author Contributions

BP searched the literature, wrote the manuscript, and constructed one of the figures. TS reviewed the first and final versions, added the segments, and constructed one of the figures. H-CH conceived this study, planned the organization of the manuscript, searched the literature, and contributed to the writing.

## Conflict of Interest Statement

The authors declare that the research was conducted in the absence of any commercial or financial relationships that could be construed as a potential conflict of interest.
